# Case of Ligature of the Primitive Carotid Artery, for the Cure of an Erectile Tumour of the Orbit, with Experiments upon Animals, to Exhibit the Influence of the Two Carotids

**Published:** 1841-01-16

**Authors:** 


### BIBLIOGRAPHICAL NOTICE.

*Case of Ligature of the Primitive Carotid Artery, for the cure of an Erectile Tumour of the Orbit, with experiments upon Animals, to exhibit the influence of the two Carotids.* By M. Jobert de Lamballe, Surgeon of the Hospital St. Louis. Report of MM. Larrey, Berard, and Gimelle, Committee of the Royal Academy of Medicine.

The number of the Bulletin of the Academy just received, contains the report of some cases of ligature of the primitive carotid, by M. Jobert, which we think it worth while to condense for the benefit of our readers. The experiments upon animals, by which they are followed, throw much light upon the changes which succeed this operation, and confirm the idea entertained by Larrey, that one of the principal dangers to be apprehended after the ligature of a large trunk is the reflux of blood to the heart, and consequent congestion or apoplexy of the lungs. The report is also interesting as giving two additional cases of the efficacy of the ligature of the primitive carotid in the cure of erectile tumours of the face.

A man aged 60, strongly built, sick for three years, offered a tumour of the right orbit, which had destroyed a part of the superciliary ridge, and was rapidly increasing; in the space of a few months it spread over the frontal bone towards the frontal protuberance, and offered pulsations isochronous with those of the radial at the wrist, a manifest expansion, and that species of *susurrus* which is found in varicose aneurisms; refrigerants and acupuncture merely hastened its growth, so that in a short time it had acquired the size of a hen's egg. The right eye was forcibly projected forwards, and each motion of the eye-lids caused intolerable pain; vision on this side was lost. After the nature of the tumour had been verified by a number of distinguished surgeons, and tested by the introduction of a fine trochar from which a continuous stream of arterial blood escaped, M. Jobert tied the right primitive carotid Aug. 7, 1839; immediately the pulsations in the tumour ceased, and the excruciating pain disappeared; the pulse rose at first, but soon resumed its normal state; voice and deglutition remained unaltered; the intellectual faculties unimpaired; there was simply a slight cough, with moderate expectoration. On the 10th, the eye could be moved in every direction without pain; union by first intention took place, and at the present time (October) the eye is contained within the orbit; no trace of the ancient tumour remains, but, in exploring with the finger, the loss of substance experienced by the frontal bone is recognised; it presents the form of a cone, with the base towards the superciliary ridge, having a width of twenty-nine millimetres, and extending in height to the frontal protuberance; in efforts to cough, the skin which covers this loss of substance is slightly raised, and immediately falls again.

After the ligature, the arteries of the right side of the face offered no pulsation, but simply undulations, which were scarcely appreciable; on the left side they had acquired an undoubted development; the sound eye has preserved its transparency, and appears even to have gained additional brilliancy and vivacity.

The author next cites a case operated upon in the same way, Aug. 22, 1836, for an erectile tumour of the right temple; in this case as in the last, no alteration was observed in the cerebral functions after the operation.

The experiments of M. Mayer gave the following as the results of ligature of the carotid in animals: upon a dog; acceleration of respiration and circulation; frequent vomitings; great drowsiness; inflammation of right eyes. Upon a goat, similar phenomena, opacity of left eye; permanent contraction of pupil. Upon horses the effects are still more serious, the animal remains at first immobile, but in a few instants falls as if struck by lightning; then succeed violent convulsions, tetanic movements, and death in about three hours: in rabbits the left eye becomes insensible and immovable, in ninety-nine cases out of one hundred, it ulcerates and suppurates, while the right retains its sensibility and motions; the inverse is the case for the ears, the right loses its functions, while those of the left remain intact; death occurs about the fourth day.

The experiments of M. Jobert, which were repeated in presence of a committee from the Academy, will be found to give results entirely opposed to those just cited—the ligature of the two carotids produced no alteration in the cerebral faculties, the senses remained unimpaired, and death occurred only in those animals where, from the relative tenuity of the vertebral arteries they were unable to serve as substitutes for the carotids—the cause of death being a congestion of the lungs.

The two carotids of a rabbit were tied: no alteration in any of the functions; killed seventy-five days after the operation; both carotids were found obliterated and injected below the obliteration; vertebral arteries apparently larger than usual; the cerebral and ophthalmic of their normal size.

Both carotids were tied in eight horses. Once the pneumo-gastric nerve being cut, the animal was seized with a violent dyspnœa, the respiration became whistling, death occurred in one hour; in a second, the recurrent nerve being cut, the animal died in two hours, the respiration continuing noisy during all this period; in a third the pneumo-gastric was comprised in the ligature; similar phenomena, death in an hour and a half; in a fourth, any injury to the pneumo-gastric was cautiously avoided, the animal exhibited no nervous symptoms, the respiration became more slow, but occurred without noise, and life was preserved six hours. A fifth, operated upon with the same precaution, experienced at the end of three hours a strong contraction of the posterior limbs, causing him to fall; he lived seven hours. The sixth lived four days. The seventh and eighth, both weakened by previous bleeding, died, one in fourteen, the other in ten hours. All were examined after death; in none did the brain present the slightest alteration, but in all, the lungs, were found greatly congested, the blood being collected in masses; less marked in the seventh and eighth, which had been bled largely before the operation.

The two primitive carotids were tied in a sheep. No unfavourable symptoms, no alteration of functions, killed twelve days afterwards: in two small dogs; no change in any function, killed in twenty-one days: in two large dogs; nothing indicated any important change in the circulation; they were killed the 25th day. The autopsy showed that in the last four the carotids had increased in size below the ligatures, were obliterated above them, and that the vertebral arteries had attained the size of the primitive carotids.

In presence of a committee of the Academy the two carotids were tied in a mule of middle size: the pneumo-gastric nerves being untouched, while the recurrents were denuded and irritated; the moment the ligatures were tightened, the respiration became whistling, and very difficult; the mouth remained half opened, as if to give passage to a greater quantity of air; when released, the animal rose, returned to his stall, and obeyed the commands of his driver to turn to the right or left; hay was offered to him, which he smelt, and afterwards masticated several times; in half an hour water was presented, of which he swallowed several mouthfuls; several times he fell backwards; the respiration become more slow, and in forty minutes he died. The autopsy exhibited the lungs gorged with blood; when incised this liquid flowed freely from every part; circumscribed extravasations existed also beneath the serous membrane.

The same operation was afterwards performed upon three horses; the nerves of the 8th pair and the recurrents were avoided with great care; the changes in respiration were less prompt and less grave than in the mule; this function was performed with difficulty, but without noise; like the mule, all these animals rose, returned to their stalls, and obeyed the directions of their masters; at the end of three-quarters of an hour they ate and drank; one lived eight hours and a half, the second thirteen hours and a quarter, and the most feeble of all lived twenty-seven hours.

The autopsy exhibited the same alterations of the lung which had been found in the mule, but here the blood was contained in veritable pouches: in the one which had survived twenty-seven hours, no portion of the lung would float in water.

In two horses the pneumogastric nerves were comprised in the ligature, the moment it was tied they uttered cries; the respiration became slow, the flanks were strongly agitated, the chest expanded with difficulty. These horses, however, as in the preceding cases, preserved all their faculties, obeying the voice and gestures of their masters. At the end of twenty minutes one fell backwards, but rose again; at the end of forty-five minutes the other did the same: one died in two hours and a half, the other in three hours and three-quarters after the operation; the same anatomical results were found as in the preceding cases.

The two carotids were tied, in two dogs, a rabbit and a sheep, Aug. 29.—These animals still live. (October.) None of them appeared to suffer much after the operation; they ate and drank as during health; the dogs barked at persons entering the room, and at present the nose, the eyes, and the ears of these animals are in a state of perfect integrity. They present none of the lesions announced by M. Mayer.

From these facts the Committee of the Acadamy confirm the conclusions of M. Jobert, which are,

1. That the ligature of the two primitive carotids in animals produces no lesion of the cerebral faculties.

2. That all the accidents bear upon the organs of respiration.

3. That death, which is the constant result in horses, arises from the congestion of the lungs, which may be said to be transformed into a pouch of blood.

4. That dogs, rabbits, and sheep, preserve their senses after this ligature.

Seventy-six cases of ligature of the primitive carotids in man are upon record; of these four were for erectile tumours developed in the orbit or eyelids, in all as in the two just added by M. J. The operation was successful. Both carotids have even been tied in the human subject by Dr. Mussey, at twelve days interval for an erectile tumour of the scalp, the success, however, was but temporary; it was found necessary afterwards to extirpate the tumour itself.

Out of these seventy-six cases observed in man, two individuals only appear to have died from a change in the cerebral circulation—a woman operated upon by Mr. Key, and a man operated upon by Lagnenbeck. Three had confusion, or dimness of vision, on the side of the artery which was tied; in two of them the accidents were of short duration; in the third the sight of the left eye was lost; five were attacked with hemiplegia of the side opposed to that upon which the ligature had been placed, of whom three recovered the use of their limbs.

A few fainted upon tightening the ligature; others when attacked with a sudden coughing; and in one case reported by Dr. Horner, loss of voice was the consequence.

These seventy-six cases do not really include all that are upon record. Some operations performed in this country, which present considerable interest, as modifying the results given by M. Jobert, have been unintentionally omitted. We believe that Dr. Mott tied the two primitive carotids in a man on the same day: and in a case operated upon by Dr. Randolph, the death of the patient, as well as the symptoms exhibited during life, were entirely referable to an affection of the brain; the right primitive carotid was tied, the patient died comatose, and the autopsy exhibited a reddish colour of the cortical substance of the left side, with a marked injection of the whole corresponding hemisphere, while the lungs presented only a few apoplectic spots. In Dr. Horner's case also, which is cited by Mons. J., it may be well to mention that the pneumo-gastric nerve was included in the ligature, and that the voice was only temporarily lost, and is now completely restored.

We have already hinted that the idea of pulmonary apoplexy as a cause of death, although corroborated by the experiments of M. Jobert, is not original with him. Larrey, in reporting upon an operation of M. Colson, by the method of Brasdor, notes, among its dangers, the reflux of blood to the left ventricle, and considers that its accumulation in this cavity will prevent the entrance of the same fluid from the corresponding auricles; a stasis will then be produced in the pulmonary veins, and these, being unprovided with valves, can readily regurgitate into the lungs, and cause apoplexy. He further states that this phenomenon (the reflux of arterial blood) was recognised by him in 1793, and that he has many times seen amputations of shoulder and thigh, followed immediately by apoplectic symptoms, which were only prevented from terminating fatally, by large general bleedings.

The high surgical reputation of M. Jobert, and the entire concurrence of a committee of the Academy in his conclusions, render this report, we think, well worthy of republication.

